# 1-{(*Z*)-1-(2,4-Dichloro­phen­yl)-1-[2-(4-methyl­phen­oxy)eth­oxy]prop-1-en-2-yl}-1*H*-imidazol-3-ium nitrate

**DOI:** 10.1107/S1600536812046181

**Published:** 2012-12-05

**Authors:** Song Guo, Yong-hong Hu, Yuan-yuan Luan, Lu-lu Wang, Wen-ge Yang

**Affiliations:** a Jiangsu Engineering Technology Research Center of Polypeptide Pharmaceutical, College of Biotechnology and Pharmaceutical Engineering, Nanjing University of Technology, Xinmofan Road No. 5 Nanjing, Nanjing 210009, People’s Republic of China; b College of Pharmaceutical Science, Nanjing University of Technology, Xinmofan Road No. 5 Nanjing, Nanjing 210009, People’s Republic of China

## Abstract

In the title salt, C_21_H_21_Cl_2_N_2_O_2_^+^·NO_3_^−^, the imidazole ring makes dihedral angles of 43.39 (14) and 10.9 (2)° with the 4-methyl­phenyl and 2,4-dichloro­phenyl rings, respectively. The mol­ecule adopts a *Z* conformation about the C=C double bond, which links the imidazole ring to the 4-methyl­phen­oxy unit *via* an eth­oxy chain. In the crystal, cations and anions are linked into chains by N—H⋯O and C—H⋯O hydrogen bonds.

## Related literature

For background to azole derivatives and synthetic details, see: Jeu *et al.* (2003 ▶); Fromtling & Castaner (1996 ▶); Ludwig & Kurt (1985 ▶). For a related structure, see: Kurt *et al.* (1987 ▶).
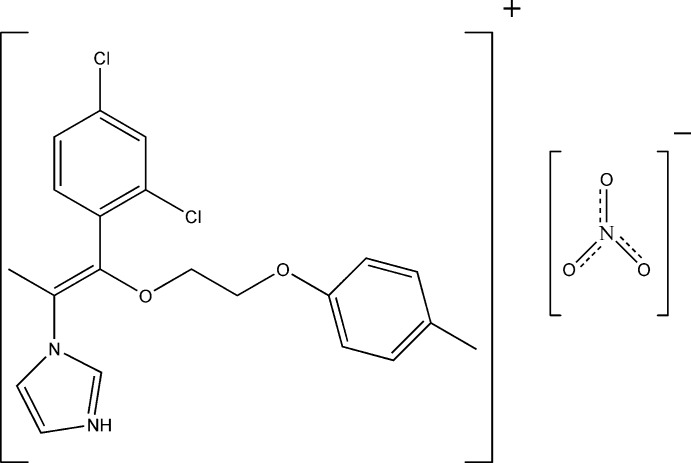


## Experimental

### 

#### Crystal data

C_21_H_21_Cl_2_N_2_O_2_^+^·NO_3_^−^
*M* *_r_* = 466.31
Triclinic, 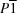

*a* = 9.5300 (19) Å
*b* = 9.924 (2) Å
*c* = 12.449 (3) Å
α = 69.05 (3)°
β = 80.59 (3)°
γ = 88.75 (3)°
*V* = 1083.9 (4) Å^3^
*Z* = 2
Mo *K*α radiation
μ = 0.34 mm^−1^
*T* = 293 K
0.30 × 0.20 × 0.10 mm

#### Data collection

Enraf–Nonius CAD-4 diffractometer
Absorption correction: ψ scan (North *et al.*, 1968 ▶) *T*_min_ = 0.905, *T*_max_ = 0.967
4247 measured reflections
3988 independent reflections
2974 reflections with *I* > 2σ(*I*)
*R*_int_ = 0.020
3 standard reflections every 200 reflections intensity decay: 1%

#### Refinement

*R*[*F* ^2^ > 2σ(*F* ^2^)] = 0.048
*wR*(*F* ^2^) = 0.158
*S* = 1.01
3988 reflections
281 parameters
H-atom parameters constrained
Δρ_max_ = 0.33 e Å^−3^
Δρ_min_ = −0.25 e Å^−3^

### 

Data collection: *CAD-4 EXPRESS* (Enraf–Nonius, 1994 ▶); cell refinement: *CAD-4 EXPRESS*; data reduction: *XCAD4* (Harms & Wocadlo, 1995 ▶); program(s) used to solve structure: *SHELXS97* (Sheldrick, 2008 ▶); program(s) used to refine structure: *SHELXL97* (Sheldrick, 2008 ▶); molecular graphics: *SHELXTL* (Sheldrick, 2008 ▶); software used to prepare material for publication: *PLATON* (Spek, 2009 ▶).

## Supplementary Material

Crystal structure: contains datablock(s) global, I. DOI: 10.1107/S1600536812046181/pv2548sup1.cif

Structure factors: contains datablock(s) I. DOI: 10.1107/S1600536812046181/pv2548Isup2.hkl

Supplementary material file. DOI: 10.1107/S1600536812046181/pv2548Isup3.cml

Additional supplementary materials:  crystallographic information; 3D view; checkCIF report

## Figures and Tables

**Table 1:** Hydrogen-bond geometry (Å, °)

*D*—H⋯*A*	*D*—H	H⋯*A*	*D*⋯*A*	*D*—H⋯*A*
N2—H2*A*⋯O4	0.86	2.55	3.218 (5)	135
N2—H2*A*⋯O5^i^	0.86	1.85	2.703 (5)	170
C13—H13*A*⋯O5^ii^	0.93	2.37	3.188 (5)	147
C21—H21*A*⋯O3^ii^	0.93	2.46	3.337 (5)	157

Symmetry codes: (i) 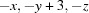; (ii) 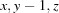.

**Fig. 1.**
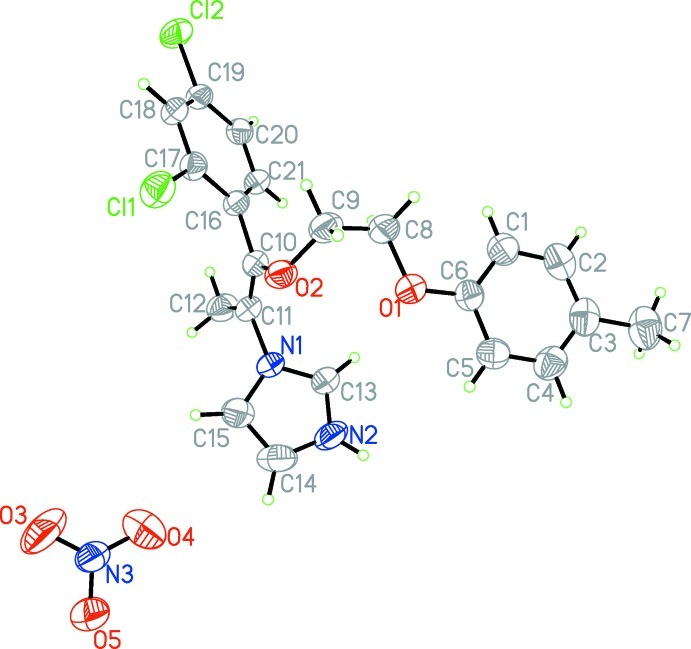
The molecular structure of the title molecule, with the atom numbering scheme. Displacement ellipsoids are drawn at 30% probability levels.

**Fig. 2.**
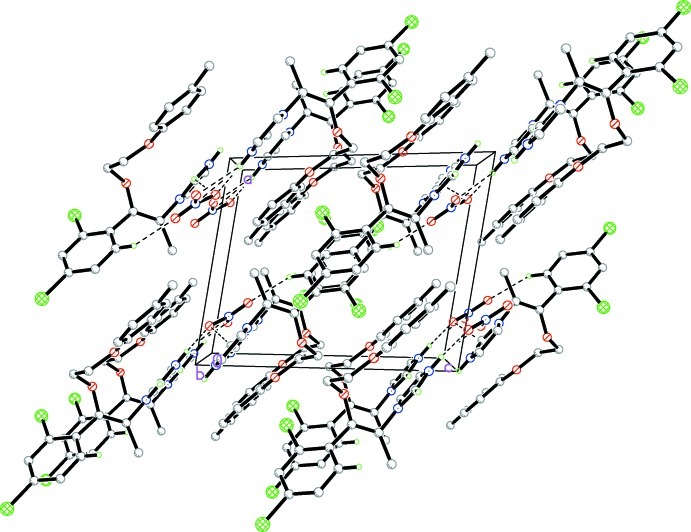
The packing diagram of the title compound. Hydron bonds are shown as dashed lines.

## supplementary crystallographic information

### Comment

Azole derivatives such as Voriconazole and Ketoconazole are safe and effective antifungal agents (Jeu *et al.*, 2003; Fromtling & Castaner,1996). As part of our studies on the synthesis of new azole derivatives, the crystal structure of the title compound was determined.

In the molecular structure of the title compound (Fig. 1) the double bond is Z configurated. In the crystal structure the anions and cations are connected via N—H···O and C—H···O hydrogen bonding (Table 1 and Fig. 2). The bond lengths and bpnd angles in the title compound agree with the corresponding bond lengths and angles reported for a closely related compound (Kurt *et al.*, 1987).

### Experimental

1-(2,4-Dichlorophenyl)-2-(1*H*-imidazol-1-yl)-1-propanone (16.6 g, 0.06 mol), 30% aqueous sodium hydroxide (50 ml), toluene (100 ml) and tetrabutylammonium hydroxide (0.26g, 0.001mol) were mixed and heated to 343.15 K under vigorous stirring. 1-(2-Bromoethoxy)-4-methyl-benzene (13.3 g, 0.06 mol), dissolved in toluene (70 ml), wais instilled into the stirred and warmed solution in the course of 10 h. The mixture was stirred at room temperature, and monitored by TLC until the reaction was complete. The reaction mixture was mixed with as much water and chloroform so that the aqueous phase becomes lighter than the organic phase. Thereafter, the organic and aqueous phases were separated. The organic phase was dried with sodium sulfate. The solvents were distilled under reduced pressure. The remaining residue was a dark oil that is diluted with 100 ml ethanol and then adjusted to a pH-value of 2 by means of 65% aqueous nitric acid. The derived nitric acid solution was cooled in the refrigerator. The impure precipitated product herein was subsequently crystallized from isopropanol. The purified product was analytically identified as an approximately pure *Z*-isomer of propylene nitrate. Crystals of title compound suitable for X-ray diffraction were obtained by slow evaporation of an ethanol solution. Details on the synthesis can be found in the literature (Ludwig & Kurt, 1985).

### Refinement

H atoms were positioned geometrically with C—H = 0.93, 0.96 and 0.97 Å for aryl, methyl and methylene H atoms, respectively, and with N—H = 0.86 Å for imidazole H atom, and constrained to ride on their parent atoms, with *U*_iso_(H) = 1.5*U*_eq_ methyl C or 1.2*U*_eq_ non-methyl C/N).

### Crystal data

**Table:**

C_21_H_21_Cl_2_N_2_O_2_^+^·NO_3_^−^	*Z* = 2
*M**_r_* = 466.31	*F*(000) = 484
Triclinic, *P*1	*D*_x_ = 1.429 Mg m^−^^3^
Hall symbol: -P 1	Mo *K*α radiation, λ = 0.71073 Å
*a* = 9.5300 (19) Å	Cell parameters from 25 reflections
*b* = 9.924 (2) Å	θ = 10–13°
*c* = 12.449 (3) Å	µ = 0.34 mm^−^^1^
α = 69.05 (3)°	*T* = 293 K
β = 80.59 (3)°	Plate, colorless
γ = 88.75 (3)°	0.30 × 0.20 × 0.10 mm
*V* = 1083.9 (4) Å^3^	

### Data collection

**Table:**

Enraf–Nonius CAD-4 diffractometer	2974 reflections with *I* > 2σ(*I*)
Radiation source: fine-focus sealed tube	*R*_int_ = 0.020
Graphite monochromator	θ_max_ = 25.4°, θ_min_ = 1.8°
ω/2θ scans	*h* = 0→11
Absorption correction: ψ scan (North *et al.*, 1968)	*k* = −11→11
*T*_min_ = 0.905, *T*_max_ = 0.967	*l* = −14→15
4247 measured reflections	3 standard reflections every 200 reflections
3988 independent reflections	intensity decay: 1%

### Refinement

**Table:**

Refinement on *F*^2^	Secondary atom site location: difference Fourier map
Least-squares matrix: full	Hydrogen site location: inferred from neighbouring sites
*R*[*F*^2^ > 2σ(*F*^2^)] = 0.048	H-atom parameters constrained
*wR*(*F*^2^) = 0.158	*w* = 1/[σ^2^(*F*_o_^2^) + (0.1*P*)^2^ + 0.230*P*] where *P* = (*F*_o_^2^ + 2*F*_c_^2^)/3
*S* = 1.01	(Δ/σ)_max_ < 0.001
3988 reflections	Δρ_max_ = 0.33 e Å^−^^3^
281 parameters	Δρ_min_ = −0.25 e Å^−^^3^
0 restraints	Extinction correction: *SHELXL97* (Sheldrick, 2008), Fc^*^=kFc[1+0.001xFc^2^λ^3^/sin(2θ)]^-1/4^
Primary atom site location: structure-invariant direct methods	Extinction coefficient: 0.034 (5)

### Special details

**Table:**

Geometry. All esds (except the esd in the dihedral angle between two l.s. planes) are estimated using the full covariance matrix. The cell esds are taken into account individually in the estimation of esds in distances, angles and torsion angles; correlations between esds in cell parameters are only used when they are defined by crystal symmetry. An approximate (isotropic) treatment of cell esds is used for estimating esds involving l.s. planes.
Refinement. Refinement of F^2^ against ALL reflections. The weighted R-factor wR and goodness of fit S are based on F^2^, conventional R-factors R are based on F, with F set to zero for negative F^2^. The threshold expression of F^2^ > 2sigma(F^2^) is used only for calculating R-factors(gt) etc. and is not relevant to the choice of reflections for refinement. R-factors based on F^2^ are statistically about twice as large as those based on F, and R- factors based on ALL data will be even larger.

### Fractional atomic coordinates and isotropic or equivalent isotropic displacement parameters (Å^2^)

**Table:**

	*x*	*y*	*z*	*U*_iso_*/*U*_eq_	
Cl1	0.27013 (8)	0.96396 (8)	0.60041 (6)	0.0542 (3)	
N1	0.1889 (2)	1.1363 (2)	0.18787 (18)	0.0431 (5)	
O1	−0.0643 (2)	0.8053 (2)	0.31896 (19)	0.0589 (6)	
C1	−0.1250 (3)	0.5853 (3)	0.2860 (3)	0.0576 (8)	
H1A	−0.0639	0.5293	0.3338	0.069*	
Cl2	0.67621 (8)	0.57854 (8)	0.62094 (7)	0.0624 (3)	
O2	0.11483 (19)	0.95339 (19)	0.40364 (16)	0.0462 (5)	
N2	0.0151 (3)	1.1943 (3)	0.0941 (2)	0.0588 (7)	
H2A	−0.0525	1.1890	0.0576	0.071*	
C2	−0.2091 (4)	0.5242 (4)	0.2333 (3)	0.0633 (9)	
H2B	−0.2039	0.4258	0.2471	0.076*	
C3	−0.3003 (3)	0.6038 (4)	0.1610 (3)	0.0587 (8)	
C4	−0.3025 (3)	0.7508 (4)	0.1395 (3)	0.0587 (8)	
H4A	−0.3605	0.8075	0.0889	0.070*	
C5	−0.2213 (3)	0.8148 (3)	0.1909 (2)	0.0548 (7)	
H5A	−0.2250	0.9136	0.1752	0.066*	
C6	−0.1338 (3)	0.7322 (3)	0.2662 (2)	0.0487 (7)	
C7	−0.3924 (4)	0.5339 (5)	0.1058 (3)	0.0797 (11)	
H7A	−0.3769	0.4322	0.1308	0.120*	
H7B	−0.3679	0.5761	0.0224	0.120*	
H7C	−0.4907	0.5490	0.1291	0.120*	
C8	0.0226 (3)	0.7263 (3)	0.4017 (3)	0.0547 (7)	
H8A	0.1129	0.7076	0.3618	0.066*	
H8B	−0.0244	0.6345	0.4519	0.066*	
C9	0.0465 (3)	0.8152 (3)	0.4726 (2)	0.0506 (7)	
H9A	−0.0444	0.8294	0.5142	0.061*	
H9B	0.1047	0.7625	0.5300	0.061*	
C10	0.2547 (3)	0.9564 (3)	0.3550 (2)	0.0398 (6)	
C11	0.2977 (3)	1.0517 (3)	0.2472 (2)	0.0432 (6)	
C12	0.4455 (3)	1.0837 (3)	0.1785 (3)	0.0577 (8)	
H12A	0.5109	1.0240	0.2241	0.087*	
H12B	0.4715	1.1835	0.1593	0.087*	
H12C	0.4487	1.0641	0.1080	0.087*	
C13	0.1000 (3)	1.0907 (3)	0.1358 (3)	0.0541 (7)	
H13A	0.0983	0.9999	0.1300	0.065*	
C14	0.0496 (4)	1.3107 (4)	0.1171 (3)	0.0654 (9)	
H14A	0.0060	1.3989	0.0956	0.078*	
C15	0.1586 (4)	1.2761 (3)	0.1766 (3)	0.0625 (9)	
H15A	0.2046	1.3352	0.2047	0.075*	
C16	0.3537 (3)	0.8592 (3)	0.4249 (2)	0.0384 (6)	
C17	0.3715 (3)	0.8572 (3)	0.5351 (2)	0.0399 (6)	
C18	0.4695 (3)	0.7714 (3)	0.5946 (2)	0.0437 (6)	
H18A	0.4807	0.7714	0.6675	0.052*	
C19	0.5516 (3)	0.6850 (3)	0.5453 (2)	0.0434 (6)	
C20	0.5350 (3)	0.6810 (3)	0.4384 (2)	0.0443 (6)	
H20A	0.5890	0.6210	0.4066	0.053*	
C21	0.4362 (3)	0.7683 (3)	0.3798 (2)	0.0420 (6)	
H21A	0.4244	0.7662	0.3077	0.050*	
O3	0.2956 (5)	1.7477 (5)	0.1574 (4)	0.1483 (17)	
O4	0.1936 (4)	1.6269 (3)	0.0810 (4)	0.1205 (12)	
O5	0.1832 (4)	1.8521 (3)	0.0225 (3)	0.1028 (10)	
N3	0.2273 (3)	1.7407 (3)	0.0876 (3)	0.0646 (7)	

### Atomic displacement parameters (Å^2^)

**Table:**

	*U*^11^	*U*^22^	*U*^33^	*U*^12^	*U*^13^	*U*^23^
Cl1	0.0563 (5)	0.0623 (5)	0.0526 (4)	0.0116 (3)	−0.0167 (3)	−0.0281 (3)
N1	0.0496 (13)	0.0425 (12)	0.0375 (11)	0.0039 (10)	−0.0188 (10)	−0.0097 (9)
O1	0.0617 (13)	0.0463 (11)	0.0704 (14)	0.0036 (9)	−0.0316 (11)	−0.0140 (10)
C1	0.0519 (17)	0.0528 (17)	0.0664 (19)	0.0028 (14)	−0.0114 (15)	−0.0185 (15)
Cl2	0.0602 (5)	0.0627 (5)	0.0686 (5)	0.0179 (4)	−0.0361 (4)	−0.0185 (4)
O2	0.0403 (10)	0.0456 (10)	0.0481 (11)	0.0047 (8)	−0.0132 (8)	−0.0091 (8)
N2	0.0570 (15)	0.0665 (17)	0.0516 (14)	0.0088 (13)	−0.0288 (12)	−0.0115 (12)
C2	0.062 (2)	0.0557 (18)	0.072 (2)	−0.0064 (15)	−0.0016 (17)	−0.0259 (16)
C3	0.0560 (18)	0.076 (2)	0.0436 (16)	−0.0140 (16)	0.0016 (14)	−0.0245 (15)
C4	0.0570 (18)	0.073 (2)	0.0422 (16)	−0.0013 (15)	−0.0117 (14)	−0.0145 (15)
C5	0.0602 (18)	0.0526 (17)	0.0463 (16)	−0.0009 (14)	−0.0117 (14)	−0.0100 (13)
C6	0.0444 (15)	0.0483 (16)	0.0500 (16)	−0.0040 (12)	−0.0079 (13)	−0.0132 (13)
C7	0.086 (3)	0.098 (3)	0.059 (2)	−0.025 (2)	−0.0081 (19)	−0.033 (2)
C8	0.0440 (16)	0.0483 (16)	0.0664 (19)	0.0031 (13)	−0.0213 (14)	−0.0090 (14)
C9	0.0403 (14)	0.0547 (16)	0.0480 (16)	0.0000 (12)	−0.0117 (12)	−0.0059 (13)
C10	0.0415 (14)	0.0390 (13)	0.0423 (14)	0.0035 (11)	−0.0152 (11)	−0.0154 (11)
C11	0.0445 (15)	0.0440 (14)	0.0428 (14)	0.0037 (11)	−0.0184 (12)	−0.0129 (12)
C12	0.0531 (17)	0.0618 (18)	0.0483 (16)	−0.0021 (14)	−0.0115 (14)	−0.0061 (14)
C13	0.0642 (19)	0.0470 (16)	0.0542 (17)	0.0030 (14)	−0.0301 (15)	−0.0131 (13)
C14	0.081 (2)	0.0560 (19)	0.0578 (19)	0.0255 (17)	−0.0242 (17)	−0.0149 (15)
C15	0.084 (2)	0.0486 (17)	0.064 (2)	0.0142 (16)	−0.0320 (18)	−0.0232 (15)
C16	0.0379 (13)	0.0397 (13)	0.0357 (13)	−0.0011 (11)	−0.0113 (10)	−0.0089 (11)
C17	0.0385 (13)	0.0411 (13)	0.0395 (14)	−0.0018 (11)	−0.0076 (11)	−0.0133 (11)
C18	0.0478 (15)	0.0456 (14)	0.0377 (14)	−0.0022 (12)	−0.0159 (12)	−0.0108 (12)
C19	0.0393 (14)	0.0410 (13)	0.0462 (15)	−0.0006 (11)	−0.0165 (12)	−0.0070 (11)
C20	0.0430 (14)	0.0423 (14)	0.0457 (15)	0.0013 (11)	−0.0090 (12)	−0.0128 (12)
C21	0.0481 (15)	0.0426 (14)	0.0360 (13)	0.0014 (12)	−0.0134 (11)	−0.0120 (11)
O3	0.183 (4)	0.180 (4)	0.144 (3)	0.071 (3)	−0.129 (3)	−0.092 (3)
O4	0.135 (3)	0.0709 (19)	0.176 (4)	0.0274 (18)	−0.062 (3)	−0.055 (2)
O5	0.128 (2)	0.0620 (15)	0.127 (2)	0.0147 (16)	−0.085 (2)	−0.0174 (16)
N3	0.0731 (18)	0.0680 (18)	0.0660 (17)	0.0236 (14)	−0.0354 (15)	−0.0309 (15)

### Geometric parameters (Å, º)

**Table:**

Cl1—C17	1.741 (3)	C8—H8A	0.9700
N1—C13	1.322 (3)	C8—H8B	0.9700
N1—C15	1.373 (4)	C9—H9A	0.9700
N1—C11	1.447 (3)	C9—H9B	0.9700
O1—C6	1.379 (3)	C10—C11	1.339 (4)
O1—C8	1.427 (3)	C10—C16	1.486 (3)
C1—C2	1.385 (4)	C11—C12	1.500 (4)
C1—C6	1.391 (4)	C12—H12A	0.9600
C1—H1A	0.9300	C12—H12B	0.9600
Cl2—C19	1.736 (3)	C12—H12C	0.9600
O2—C10	1.367 (3)	C13—H13A	0.9300
O2—C9	1.434 (3)	C14—C15	1.342 (5)
N2—C13	1.304 (4)	C14—H14A	0.9300
N2—C14	1.344 (4)	C15—H15A	0.9300
N2—H2A	0.8600	C16—C21	1.391 (4)
C2—C3	1.381 (5)	C16—C17	1.403 (4)
C2—H2B	0.9300	C17—C18	1.374 (4)
C3—C4	1.386 (5)	C18—C19	1.383 (4)
C3—C7	1.513 (5)	C18—H18A	0.9300
C4—C5	1.374 (4)	C19—C20	1.380 (4)
C4—H4A	0.9300	C20—C21	1.381 (4)
C5—C6	1.386 (4)	C20—H20A	0.9300
C5—H5A	0.9300	C21—H21A	0.9300
C7—H7A	0.9600	O3—N3	1.189 (4)
C7—H7B	0.9600	O4—N3	1.215 (4)
C7—H7C	0.9600	O5—N3	1.227 (4)
C8—C9	1.496 (4)		
			
C13—N1—C15	108.1 (2)	H9A—C9—H9B	107.8
C13—N1—C11	125.4 (2)	C11—C10—O2	118.1 (2)
C15—N1—C11	126.5 (2)	C11—C10—C16	122.7 (2)
C6—O1—C8	118.6 (2)	O2—C10—C16	119.1 (2)
C2—C1—C6	118.8 (3)	C10—C11—N1	116.8 (2)
C2—C1—H1A	120.6	C10—C11—C12	128.5 (2)
C6—C1—H1A	120.6	N1—C11—C12	114.8 (2)
C10—O2—C9	117.7 (2)	C11—C12—H12A	109.5
C13—N2—C14	109.5 (3)	C11—C12—H12B	109.5
C13—N2—H2A	125.3	H12A—C12—H12B	109.5
C14—N2—H2A	125.3	C11—C12—H12C	109.5
C3—C2—C1	122.4 (3)	H12A—C12—H12C	109.5
C3—C2—H2B	118.8	H12B—C12—H12C	109.5
C1—C2—H2B	118.8	N2—C13—N1	108.5 (3)
C2—C3—C4	117.4 (3)	N2—C13—H13A	125.7
C2—C3—C7	121.3 (3)	N1—C13—H13A	125.7
C4—C3—C7	121.3 (3)	C15—C14—N2	107.5 (3)
C5—C4—C3	121.7 (3)	C15—C14—H14A	126.3
C5—C4—H4A	119.2	N2—C14—H14A	126.3
C3—C4—H4A	119.2	C14—C15—N1	106.4 (3)
C4—C5—C6	120.0 (3)	C14—C15—H15A	126.8
C4—C5—H5A	120.0	N1—C15—H15A	126.8
C6—C5—H5A	120.0	C21—C16—C17	117.5 (2)
O1—C6—C5	115.3 (2)	C21—C16—C10	119.8 (2)
O1—C6—C1	125.1 (3)	C17—C16—C10	122.7 (2)
C5—C6—C1	119.6 (3)	C18—C17—C16	121.1 (2)
C3—C7—H7A	109.5	C18—C17—Cl1	118.0 (2)
C3—C7—H7B	109.5	C16—C17—Cl1	120.9 (2)
H7A—C7—H7B	109.5	C17—C18—C19	119.5 (2)
C3—C7—H7C	109.5	C17—C18—H18A	120.3
H7A—C7—H7C	109.5	C19—C18—H18A	120.3
H7B—C7—H7C	109.5	C20—C19—C18	121.3 (2)
O1—C8—C9	107.8 (2)	C20—C19—Cl2	119.8 (2)
O1—C8—H8A	110.1	C18—C19—Cl2	118.9 (2)
C9—C8—H8A	110.1	C19—C20—C21	118.3 (2)
O1—C8—H8B	110.1	C19—C20—H20A	120.8
C9—C8—H8B	110.1	C21—C20—H20A	120.8
H8A—C8—H8B	108.5	C20—C21—C16	122.2 (2)
O2—C9—C8	113.2 (2)	C20—C21—H21A	118.9
O2—C9—H9A	108.9	C16—C21—H21A	118.9
C8—C9—H9A	108.9	O3—N3—O4	122.8 (4)
O2—C9—H9B	108.9	O3—N3—O5	119.1 (3)
C8—C9—H9B	108.9	O4—N3—O5	118.0 (3)
			
C6—C1—C2—C3	0.6 (5)	C14—N2—C13—N1	1.0 (4)
C1—C2—C3—C4	1.8 (5)	C15—N1—C13—N2	−0.8 (4)
C1—C2—C3—C7	−179.2 (3)	C11—N1—C13—N2	178.6 (3)
C2—C3—C4—C5	−2.2 (5)	C13—N2—C14—C15	−0.9 (4)
C7—C3—C4—C5	178.8 (3)	N2—C14—C15—N1	0.4 (4)
C3—C4—C5—C6	0.2 (5)	C13—N1—C15—C14	0.2 (4)
C8—O1—C6—C5	177.2 (3)	C11—N1—C15—C14	−179.2 (3)
C8—O1—C6—C1	−0.8 (4)	C11—C10—C16—C21	−56.0 (4)
C4—C5—C6—O1	−175.8 (3)	O2—C10—C16—C21	126.9 (3)
C4—C5—C6—C1	2.3 (4)	C11—C10—C16—C17	122.3 (3)
C2—C1—C6—O1	175.3 (3)	O2—C10—C16—C17	−54.9 (3)
C2—C1—C6—C5	−2.7 (4)	C21—C16—C17—C18	1.8 (4)
C6—O1—C8—C9	−162.1 (2)	C10—C16—C17—C18	−176.5 (2)
C10—O2—C9—C8	−70.2 (3)	C21—C16—C17—Cl1	−178.48 (19)
O1—C8—C9—O2	−59.3 (3)	C10—C16—C17—Cl1	3.2 (4)
C9—O2—C10—C11	141.4 (2)	C16—C17—C18—C19	−0.3 (4)
C9—O2—C10—C16	−41.2 (3)	Cl1—C17—C18—C19	179.9 (2)
O2—C10—C11—N1	−4.6 (4)	C17—C18—C19—C20	−1.3 (4)
C16—C10—C11—N1	178.2 (2)	C17—C18—C19—Cl2	179.39 (19)
O2—C10—C11—C12	175.2 (3)	C18—C19—C20—C21	1.5 (4)
C16—C10—C11—C12	−2.0 (4)	Cl2—C19—C20—C21	−179.2 (2)
C13—N1—C11—C10	−78.8 (4)	C19—C20—C21—C16	0.0 (4)
C15—N1—C11—C10	100.4 (3)	C17—C16—C21—C20	−1.6 (4)
C13—N1—C11—C12	101.3 (3)	C10—C16—C21—C20	176.7 (2)
C15—N1—C11—C12	−79.5 (4)		

### Hydrogen-bond geometry (Å, º)

**Table:**

*D*—H···*A*	*D*—H	H···*A*	*D*···*A*	*D*—H···*A*
N2—H2*A*···O4	0.86	2.55	3.218 (5)	135
N2—H2*A*···O5^i^	0.86	1.85	2.703 (5)	170
C13—H13*A*···O5^ii^	0.93	2.37	3.188 (5)	147
C21—H21*A*···O3^ii^	0.93	2.46	3.337 (5)	157

Symmetry codes: (i) −*x*, −*y*+3, −*z*; (ii) *x*, *y*−1, *z*.
